# Design of an Acoustic Through-Casing Logging Tool

**DOI:** 10.3390/s22218404

**Published:** 2022-11-01

**Authors:** Kai Zhang, Shengqing Li, Yuanda Su, Baohai Tan, Bo Zhang

**Affiliations:** 1 School of Geosciences and Technology, China University of Petroleum, Qingdao 266555, China; 2 Shuoren Times Technology, Suite 304, Rainbow Plaza, 11 Information Rd, Haidian District, Beijing 100085, China

**Keywords:** through-casing logging, acoustic logging, logging tool, transmitting transducer, transducer excitation circuit

## Abstract

Well logging is performed in oil and gas exploration wells to obtain the physical characteristics of underground formations. Thereafter, these wells are cased. Through-casing logging is important in mature fields and for wells that are cased without logging due to borehole stability issues. Acoustic through-casing logging is a challenging issue due to the strong interference of casing waves in formation waves, especially when the casing and formation are poorly bonded. An acoustic tool with dual-source transmitters is developed, in which an additional transducer is added to suppress casing waves. First, the operation principle and the overall design of the tool are carried out, including the span distance between the two transmitting transducers and the spacing distance between the transmitting transducer and the receiving transducers. Thereafter, a dual-source transmitting circuit is designed to send out two excitation signals of opposite polarities. These signals possess good consistency, high emission power, and precise signal adjustment. Lastly, the tool is tested in cased exploration wells in China. The experiment endings show that about 90% of the casing waves are canceled. By suppressing the casing wave amplitude, the cased-hole acoustic logging can be used commercially to obtain trustworthy formation information.

## 1. Introduction

In oil and gas exploration and exploitation, logging tools measure the physical parameters of the underground layers as they are lifted up along the exploration wells. These tools include resistivity, acoustic, density, neutron, and gamma-ray tools [1,2,3,4]. The fundamental principle of acoustic logging is that when acoustic signals propagate through formations, their velocities, amplitudes, and frequencies are changed with the lithological and porosity character of the formations. Compared with other logging tools, acoustic logging endings are not influenced by the characteristics and penetration of mud or the mineralization rate of the formations. Therefore, acoustic logging tools play an important role in detecting the lithology and porosity of underground formations, identifying the properties of the fluids in the formation, and recognizing the fractures of the formation [5,6]. During acoustic logging, transmitting transducers are excited by electrical excitation sources to produce acoustic waves [7,8,9]. Thereafter, these acoustic waves propagate into rock formations and are finally collected by receiving transducers. After well logging, the wells are cased to prevent communication between zones.

Given the vast number of cased exploration wells, through-casing logging is indispensable in mature fields and for wells that are cased without logging [10,11,12,13]. Through-casing resistivity and nuclear technologies have been applied commercially [14,15,16], but acoustic through-casing technology is still an unresolved issue [17]. This issue is caused by the strong interference of casing waves in the useful formation waves, especially when the formation and casing are poorly bonded [13]. In signal processing, corresponding techniques have been developed after the signal is collected to eliminate the casing waves and understand acoustic formation properties [18,19,20,21]. However, a foundation for signal processing techniques is that the collected data have an adequate signal-to-noise ratio to extract the formation information [22]. This foundation is not satisfied in a poorly bonded borehole. The reason is that the gain for the digitizer is always controlled by the casing waves of a large amplitude in data digitization during logging, resulting in the formation signal of a small amplitude being poorly digitized and hard to extract. Therefore, acoustic through-casing logging must suppress the casing waves before the digitization of analog signals.

In the process of logging in a cased well, the head waves collected by the receiving transducer are the waves propagating along the casing. As the thickness of the casing is far less than its wavelength, the casing wave is a kind of induced wave propagating as the Lamb wave. The acoustic waves radiated by the source-transducer spread into the casing with certain incidence angles; thereafter, these waves will produce multiple reflections on the different reflection points of the inner and outer walls of the casing. Meanwhile, as the reflection coefficient of the casing outer wall is a function of the physical characteristics of the media on the interface’s two sides, as well as a function of the incident angle, the casing waves will take place at different attenuations. Lastly, the casing waves received by array receivers are the superposition of the first reflected wave and the multiple reflected waves in the casing.

To measure the acoustic properties of through-casing formations, an acoustic tool with dual-source transmitters was developed to suppress the casing waves. One additional transducer was added to suppress casing waves.

## 2. Operation Principle of the Dual-Source Technology

**Figure 1:**
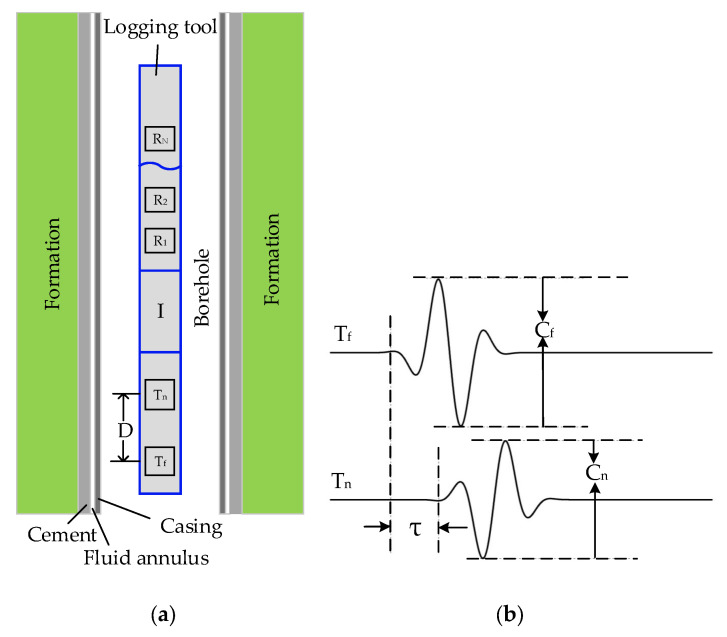
Dual-source logging tool model in the cased-hole well (**a**), and excitation signals for the far and near transmitting transducers (**b**).

The dual-source logging tool has one more excitation sound source than conventional logging tools. Figure 1 illustrates the cased-hole model for dual-source logging [23]. The acoustic tool with dual transmitters is centered in the cased borehole model. A fluid annulus between the casing and cement (or between the cement and formation) is used to symbolize the poor bonding condition. The tool consists of a receiver array of N receivers (R_1_, R_2_, …, R_N_) and two transmitters, named the near transmitter T_n_ and far transmitter T_f_, respectively. The span distance between the two transmitters is D.

During logging, the T_f_ transmitter is excited first, with an excitation amplitude C_f_. The T_n_ transmitter is then fired with an excitation amplitude C_n_ after a delay time τ. The polarities of the T_n_ and T_f_ excitation signals are opposite to each other. By adjusting the values of C_f_, C_n_, and τ accurately, the two casing waves sequentially excited by the two transmitters can cancel each other while they propagate toward the receiver array. The formation signals, whose propagation velocities are usually slower than the casing waves, will be preserved. Therefore, for a poorly bonded cased borehole, the dual transmitter acquisition method eliminates the casing waves and obtains the desired formation acoustic signals.

## 3. Overall Design of the Acoustic LWD Tool

This designed tool has one more excitation sound source, while other structures are basically the same as conventional tools [24,25]. The span distance between the two transmitting transducers and the spacing distance between the transmitting transducer and the receiving transducers must be redesigned.

### 3.1. The Spacing Distance between the Transmitting Transducer and the Receiving Transducer

In the excitation process of the driving-voltage signal, the acoustic waves radiated by the source-transducer cannot contain all the wave patterns of the longitudinal wave, shear wave, and Stoneley wave at the same time, and what is radiated is any one of these wave patterns. Assuming that the radiated wave by the source-transducer is a longitudinal wave, the radiated longitudinal wave can generate mode conversion waves during the process of their propagation and reflection/refraction in the media, such as the wave patterns of reflected and refracted longitudinal waves, reflected and refracted shear waves, and other waves induced at the interface of the media [26]. To detect the longitudinal waves and shear waves in the time domain, the spacing distance should be long enough to ensure that the head longitudinal waves appear after the follow-up shear waves. Meanwhile, a long spacing distance is beneficial to the exploration depth of the logging tool. To calculate the suitable spacing distance, the compressional wave velocity of the fastest dolomite formation is assumed to be 7900 m/s (slowness is 127 μs/m), and its shear wave velocity is 4400 m/s (slowness is 227 μs/m). Then, the slowness difference between the compressional and shear wave is 100 μs/m. To guarantee that their arrival time difference is more than two periods (200 μs), the minimum spacing distance should be greater than 2 m (the domain source frequency of the tool is 10 kHz). Meanwhile, if the source distance is too long, the received signal will be too weak to detect. Therefore, the minimum source distance should be between 2.13 m and 3.66 m.

**Figure 2:**
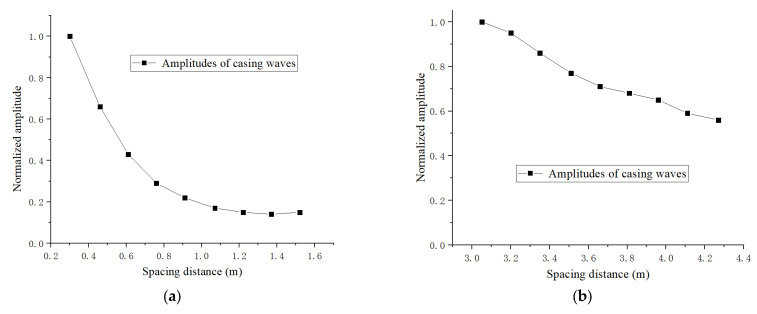
Normalized amplitudes of the casing waves excited by the same transmitting transducers and received by the same receiving transducers, while the propagation lengths are different short (**a**) and long (**b**) spacing distances.

On the other hand, as casing waves are excited by different transmitting transducers and received by the same receiving transducers, the spacing distance would also influence the amplitude matching between the casing waves excited by the far and near transmitters. To find out the effecting law, acoustic experiments were carried out to measure casing waves of different propagation lengths. In these experiments, the same transmitting transducer was excited by the same exciting circuit, while the same receiving transducer received the casing waves as the spacing distance changed. Figure 2a is the normalized amplitudes of the casing waves for the spacing distance between 0.30 m and 1.52 m, and Figure 2b is for the distance between 3.05 m and 4.27 m. When it varies from 0.30 m to 1.52 m, the amplitude decays exponentially and rapidly with the spacing distance; when the distance varies from 3.05 m to 4.27 m, the amplitude decays linearly and slowly. The reason is that: scattering attenuations occur while casing waves propagate along the case. As these waves produce multiple reflections on the different reflection points of the inner and outer walls of the casing, the attenuations of casing waves follow the negative exponential law. That is, a long spacing distance is beneficial to the amplitude matching between the two casing waves.

### 3.2. The Span Distance between the Two Transmitting Transducers

The decrease in the span distance is beneficial to eliminating the casing waves, but it will also weaken the desired formation signals. The increase in the span distance amplifies the path differences of the casing waves excited by the two transmitting transducers, leading to a reduction in their consistency. To solve this problem, numerical simulations were carried out based on the real-axis integration method, and the signal-to-noise ratios of the formation waves excited by the dual-source were quantitatively analyzed. Thereafter, the suppression coefficients of the casing waves were calculated by extracting the peak-to-peak values of the casing waves and formation waves with different dual-source span distances.

The specific calculation steps are as follows:

1. In the case of single-source excitation, the peak-to-peak value of the casing wave (C_1_) is calculated when the cased well is poorly cemented. Thereafter, the peak-to-peak value of the formation wave (F_1_) is calculated when the cased well is completely cemented.
2. Regarding the casing wave as a noise signal and the formation wave as a useful signal, their ratio (k_1_ = F_1_/C_1_) can be considered as the signal-to-noise ratio in the case of single-source excitation.
3. In the case of dual-source extraction, the peak-to-peak value of the casing wave (C_2_) is calculated when the cased well is poorly cemented. Thereafter, the peak-to-peak value of the formation wave (F_2_) is calculated when the cased well is completely cemented.
4. The signal-to-noise ratio (k_2_ = F_2_/C_2_) in the case of dual-source extraction is calculated.
5. The ratio of k_2_ to k_1_ (G = k_2_/k_1_) is calculated to reflect the casing wave suppression effect of the dual-source method.

**Figure 3:**
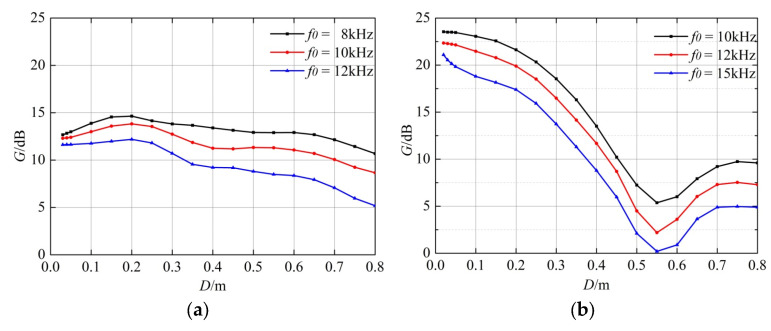
Relationship between the suppression effect of the casing wave and the span distance for a hard formation (**a**) and a soft formation (**b**).

Numerical simulations were performed using the real axis integration method to analyze the casing wave suppression effects with different dual-source span distances. The cased-hole model is shown in Figure 1, while the corresponding parameters are shown in Table 1. The well is in a poor bonding condition when the fluid annulus exists. Meanwhile, it is completely cemented when there is no fluid annulus. Figure 3 shows the casing wave suppression effects for a hard formation and a soft formation with different dual-source span distances, respectively. The black, red, and blue curves in Figure 3a are the signal-to-noise ratios for a hard formation, with the domain frequencies of the sound source being 8 kHz, 10 kHz, and 12 kHz, respectively. The black, red, and blue curves in Figure 3b are the signal-to-noise ratios for a soft formation, with the main frequencies of the sound source being 10 kHz, 12 kHz, and 15 kHz, respectively. These two figures illustrate that the suppression effects are more obvious when the frequencies are low and the span distances are short. After detailed calculation, the optimal span distance is certified to be between 0.10 m and 0.30 m.

**Figure 4:**
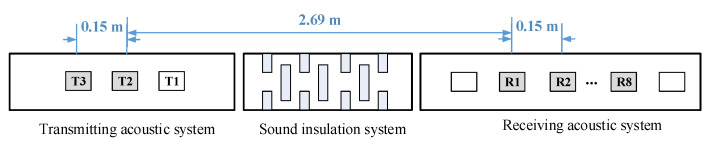
Established structural diagram of the designed sources.

When the above factors are taken into consideration, the established structural diagram of the designed sources is shown in Figure 4. For the purpose of experiments and possible adjustment, three excitation and ten reception fixing frames were designed, but only two excitation and eight reception transducers were included. These excitation and reception transducers were installed with a span distance of 0.15 m and a spacing distance of 2.69 m.

**Figure 5:**
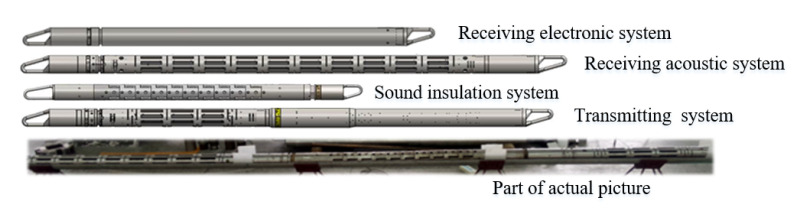
Structural diagram and part of the actual picture of the logging tool.

The whole structural diagram of the logging tool is shown in Figure 5, the through-casing sonic logging tool consists of four parts, a transmitting system, a sound insulation system, a receiving acoustic system, and a receiving electronic system. The transmitting system is mainly used to generate high-voltage signals to excite transmitting transducers, which can translate electronic signals into acoustic signals. The sound insulation system delays and attenuates the direct wave propagating along the tool. The receiving acoustic system contains eight receiving transducers, which can translate acoustic signals into electronic signals. The receiving electronic system filters and amplifies the electronic signals received by the receiving transducers and then performs quantitative acquisition and uploads the acquired data to the surface device of the logging unit.

## 4. The Design of the Dual-Source Transducer Excitation Circuit

Traditional transducer excitation circuits produce positive pulse signals of high voltage, which are not suitable for the designed dual-source tool [27,28]. First, this tool needs to carry out two excitation signals of opposite polarities and a good consistency. Second, the amplitude, frequency, and delay time of the excitation signals should be adjusted precisely. Lastly, to ensure that the sound wave can pass through the casing to reach the formation and be received by receiving transducers, the transmitters should have sufficient emission power. Therefore, a dual-source transmitting circuit is designed.

**Figure 6:**
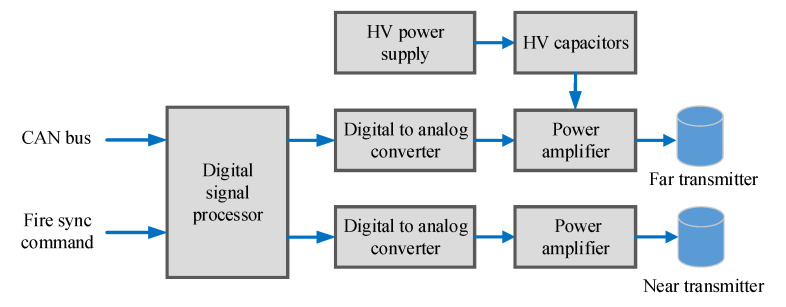
Schematic diagram of the dual-source excitation circuit.

**Figure 7:**
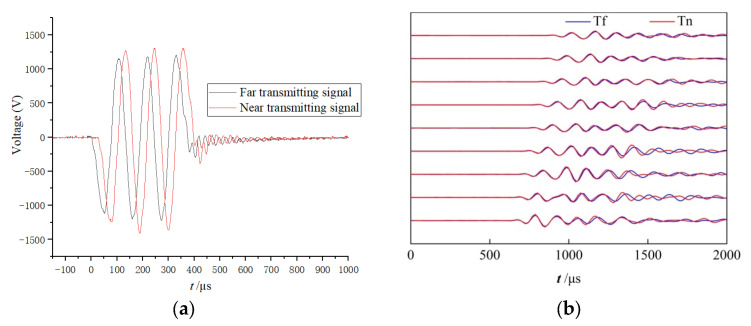
Excitation signals for the near and far excitation signals (**a**), and the reception signals of the eight receiving transducers (**b**) excited by the far and near transmitters.

The schematic diagram of the dual-source excitation circuit is shown in Figure 6. It consists of a transmitting controller, two digital-to-analog converters, two power amplifiers, a high-voltage power supply, and a series of high-voltage storage capacitors. The transmitting controller uses a high-speed digital signal processor (DSP). The workflow is as follows. First, the DSP collects control signals, which are mainly about the amplitude, polarity, frequency, and delay time of the excitation signals from the central control system by the Controller Area Network bus. Second, the DSP waits for the fire sync command generated by signal acquisition circuits. When the command comes, it acquires the wave data stored in its FLASH storage and outputs these data into the digital-to-analog converters. Third, these core circuits produce low-voltage excitation signals, which are immediately amplified by power amplifiers (a kind of class AB push–pull excitation circuit) to several thousands of volts. Lastly, high-voltage excitation signals are applied to transmitting transducers to excite acoustic signals. The frequency and amplitude of the high-voltage waveform are changed by adjusting the corresponding characteristics of the low-voltage signal output by the DAC. In our experiments, the excitation signals were designed as pulse square waves and sinusoidal waves with different circles. The pulse square waves and sinusoidal waves with few circles contain many frequency components. The sinusoidal waves with many circles contain few components, but the acoustic wave train becomes long, and the casing wave with a long duration is more easily aliased with the formation wave. Therefore, we choose the sinusoidal waves of three circles as the excitation signals, as shown in Figure 7a.

## 5. Experiments in the Exploration Well

The dual-source tool was tested in a cased exploration well in Zhongyuan Oil Field of China. Since the consistency of the casing waves excited by the far and near transmitters was very important, opposite experiments were conducted to carry out the best consistency as follows. First, the eight receiving transducers collected signals only when the far transmitter was excited. Second, the amplitude and delay time of the excitation signal for the near transmitter were adjusted. Third, the receiving transducers collected signals only when the near transmitter was excited. Lastly, these collected signals were compared, while the parameters were adjusted to fit the best consistency. After a series of tests, the optimal amplitude ratio of the far and near excitation signals was set to 1:0.90, and the delay time of the near signal was 28 μs.

Figure 7a shows the two excitation signals for the near and far excitation signals, and Figure 7b shows the reception signals of the eight receiving transducers. The blue curves (T_f_) are the waveforms received only when the far transmitter works, whereas the red curves (T_n_) are the waveforms received only when the near transmitter works. The two series of waveforms coincide well, indicating that the far and near excitation sources have a good consistency. It should be noted that the excitation signals are of the same polarity in order to test the consistency, and those are of the opposite polarity in order to perform normal logging.

**Figure 8:**
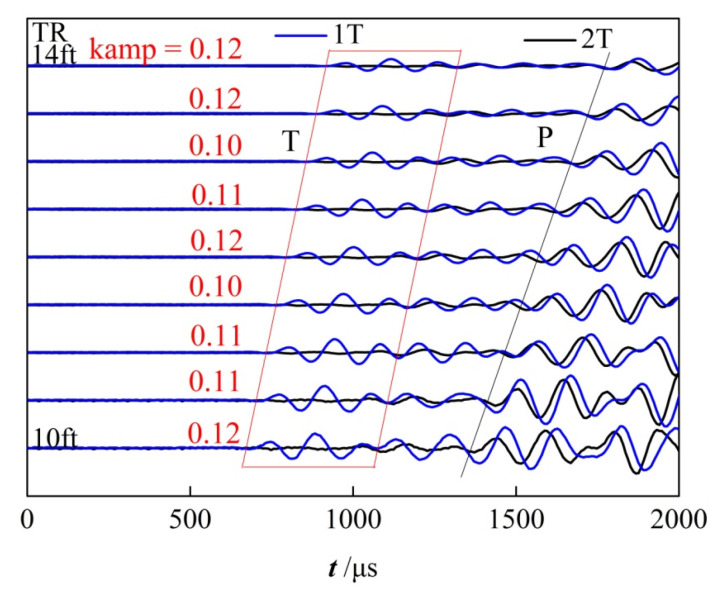
Waveforms are collected by eight receiving transducers at a certain depth, which are excited by single-source (blue) and dual-source (black), respectively.

Figure 8 shows the waveforms collected by the receiving transducers at a certain depth. The blue waveforms are collected only when the far transmitter works, while the black waveforms are collected when the dual-source transmitters work. The time window marked by the red box is the period when the casing waves arrive, and the amplitude ratios of the casing waves of the one transmitter and the dual-source transmitters are calculated and shown in red. It can be seen that the dual-source excitation method has a significant suppression effect on the casing waves, as the amplitude ratios are about 0.1~0.12. Meanwhile, the amplitudes of the subsequent formation longitudinal waves (marked by the slash P) do not change much. That is, since the casing waves are suppressed, the arrival times of the longitudinal formation waves become clear.

**Figure 9:**
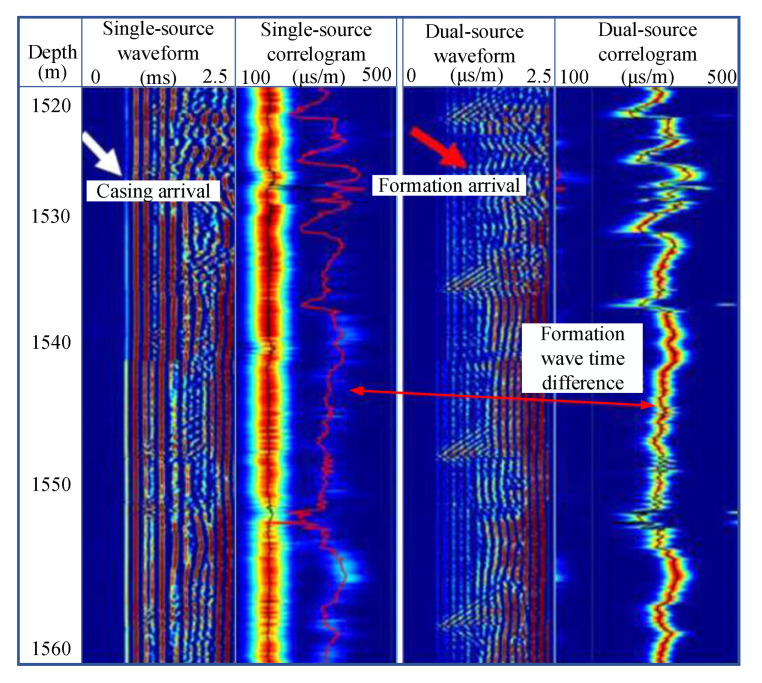
Processing results of the logging data collected by the designed tool in the depth section of 1520 m~1560 m. From left to right, the panels in the figure are the depth, the variable density log (VDL) display of the far transmitter, the Slowness–Time–Coherence (STC) correlogram of the far transmitter, the VDL display of the dual-source, and the STC correlogram of the dual source.

Figure 9 shows the processing results of the logging data in the depth section of 1520 m~1560 m. To compare the logging endings of the traditional method and the dual-source method, the logging data were collected and processed only when the far transmitter worked. Thereafter, the data were collected and processed when the dual transmitters worked. From left to right, the panels in the figure are the depth, the variable density log (VDL) display of the far transmitter, the Slowness–Time–Coherence (STC) correlogram of the far transmitter, the VDL display of the dual-source, and the STC correlogram of the dual source. The VDL display of the far transmitter shows the strong and almost invariant casing wave arrival from the beginning to the time of 1200 microseconds. The formation signal (i.e., the P-wave arrival) is overwhelmed by the casing wave and cannot be visually detected in the time section. Consequently, the STC correlogram shows a strong coherence strip with an almost constant slowness value of 190 μs/m corresponding to the casing wave slowness, together with some low-coherence events corresponding to the slowness values of the formation P-wave signal buried in the casing waves.

It is worth mentioning that the casing wave in the actual dual-transmitter measurement may not be completely canceled. The above experiments show that about 90% of the casing wave amplitudes are canceled, which means that casing waves will still exist in the data of a dual-transmitter tool. Compared with the single-source excitation logging data processing results, the casing wave is mostly suppressed with the dual-source method. The formation signal is visible, indicating that the signal-to-noise ratio has been greatly improved. The STC correlogram shows that the P-wave slowness varies with depth. As a result, the correlation of formation compressional waves is significantly improved. The slowness of formation compressional waves can be easily extracted, and the extracted formation P-wave slowness curve is displayed in the third panel of the STC correlogram of the far transmitter. It certifies that the extracted slowness curve coincides with the (weak) formation P-wave correlation signal. By suppressing the amplitude of the casing waves, the dual-source tool can now achieve adequate sampling for the formation acoustic signal in the A/D digitization processing; meanwhile, the signal-to-noise ratios of the collected data are significantly enhanced. On the other side, the remaining casing waves can now be suppressed by signal processing methods easily.

## 6. Conclusions

To realize acoustic through-casing logging, an acoustic tool with dual-source transmitters was developed, in which an additional transducer was added to suppress casing waves. The structure of the tool was basically the same as that of the conventional logging tool. The span distance between the two transmitting transducers was designed to be 0.15 m, and the spacing distance between the transmitting transducer and the receiving transducers is 2.59 m. A dual-source transmitting circuit was designed to carry out two excitation signals of opposite polarity, good consistency, and high emission power. The tool was tested in cased exploration wells; the experiment endings showed that about 90% of the casing wave amplitudes were canceled. The casing waves cannot be fully canceled. First, the casing waves excited by the far transmitter and near transmitter propagate along different paths. Meanwhile, the reflection points on the inner and outer walls of the casing are different, and the cement bonding quality of each reflection point is also different. Second, the acoustic waves radiated by the source-transducer outwardly are an acoustic signal wavelet that contains many frequency components. Both the borehole mud and casing have varying degrees of viscosity, resulting in dispersion. As a result, each frequency component travels at a different speed and produces a different attenuation in the mud and casing. Even if the two source transducers radiate the same acoustic waves outward, the casing wave reaching the receiver will be different. Third, the parameters of the transmitting transducers and their excitation circuits are not exactly the same, which would also affect the casing wave cancellation.

By suppressing the casing wave amplitude, the dual-source tool can achieve adequate sampling in the A/D digitization processing and enhance the signal-to-noise ratio of the collected data significantly. Meanwhile the remaining casing wave can now be suppressed by signal processing methods. Thereafter, the cased-hole acoustic logging can be routinely used to obtain reliable formation information through the casing.

## Figures and Tables

**Table 1:** Parameters of the cased-hole acoustic model.

Parameters	Compressional Velocity (m/s)	Shear Velocity (m/s)	Density (kg/m^3^)	Outside Diameter (m)
Fluid in the well	1500	0	1000	0.14
Casing	6098	3354	7500	0.16
Fluid annulus	1500	0	1000	0.17
Cement	2823	1729	1920	0.2
Soft formation	3000	1200	2300	∞
Hard formation	4000	2300	2500	∞

## References

[1] Wu L., Dong Z.Z., Li W.R., Jing C., Qu B.C. (2021). Well-Logging Prediction Based on Hybrid Neural Network Model. Energies.

[2] Nieto J., Badry R., Kovacs J., Ellis D., Faivre O., Mosse L. (2005). Improved density logging in hot, rugose carbonate formations. Petrophysics.

[3] Ren Z.P., Dang R.R. A New Resistivity Logging Method in Production Well. Proceedings of the 2nd International Conference on Energy, Environment and Sustainable Development (EESD 2012).

[4] Craig M. (2019). Transfer Functions for Predicting Borehole Gamma-Ray Logs. Pure Appl. Geophys..

[5] Zhang K., Tan B., Zhang W., Sun Y., Zheng J., Su Y., Liu X., Wu G., Xin S. (2021). Design of a New Acoustic Logging While Drilling Tool. Sensors.

[6] Asfahani J., Ghani B.A. (2012). Automated interpretation of nuclear and electrical well loggings for basalt characterization (case study from southern Syria). Appl. Radiat. Isot..

[7] Zhang K., Li F., Wu J., Tan B., Liu L. (2022). Pulse width research on half-sine excitation signal for bending vibrator. J. Geophys. Eng..

[8] Zhou Y., Wang X., Xin P., Dai Y. (2016). Studies on piezoelectric cylindrical transducer optimization for dipole acoustic logging. J. Appl. Acoust..

[9] Zhang K., Tan B., Liu X. (2017). A Consistency Evaluation and Calibration Method for Piezoelectric Transmitters. Sensors.

[10] Chang S.K., Everhart A.H. (1983). A Study of Sonic Logging in a Cased Borehole. J. Pet. Technol..

[11] Tubman K.M., Cheng C.H., Toksoez M.N. (1984). Synthetic full waveform acoustic logs in cased boreholes. Geophysics.

[12] Tubman K.M., Cheng C.H., Toksöz M.N. Wave Propagation In Boreholes With Unbonded Casing. Proceedings of the 1984 SEG Annual Meeting.

[13] Pampuri F., Borghi M., Deias S., Giorgioni M., Brambilla F., Tang X., Patterson D. Compressional Slowness Determination Behind Poorly-Cemented Casing (Case History). Proceedings of the Offshore Mediterranean Conference and Exhibition.

[14] Aulia K., Poernomo B., Richmond W.C., Wicaksono A.H., Beguin P.C., Benimeli D., Dubourg I. (2001). Resistivity Behind Casing. Oilfield Rev..

[15] Zhou Q., Wicaksono A. (2005). Proper interpretation of cased-hole resistivity logs. Petrophysics.

[16] Bryant I.D. (2002). Well-bore reservoir evaluation technologies to optimize field revitalization. Pet. Geosci..

[17] Moos D., Dvorkin J. (1996). Sonic logging through casing for porosity and fluid characterization in the Wilmington Field, CA. SEG Technical Program Expanded Abstracts 1996.

[18] Xiaoming T., Patterson D. Analyzing And Processing Acoustic Logging Data For Poorly-Bonded Cased Boreholes. Proceedings of the SPWLA 46th Annual Logging Symposium.

[19] Tang X.-M., Patterson D.J. (2010). Mapping formation radial shear-wave velocity variation by a constrained inversion of borehole flexural-wave dispersion data. Geophysics.

[20] Su Y.-D., Tang X.-M., Zhuang C.-X., Xu S., Zhao L. (2013). Mapping formation shear-velocity variation by inverting logging-while-drilling quadrupole-wave dispersion data. Geophysics.

[21] Murray D.R., Li Y.H., Voskamp A., Qiang L. Advanced Compressional and Shear Slowness Sonic Acquisition in Cased Wells. Proceedings of the 9th SEGJ International Symposium.

[22] Valero H.P., Skelton O., Cao H. (2001). A practical sonic slowness evaluation behind casing. SEG Technical Program Expanded Abstracts 2001.

[23] Jiang C., Chen X.-L., Su Y.-D., Tang X.-M. (2019). Cased borehole acoustic-wave propagation with varying bonding conditions: Theoretical and experimental modeling. Geophysics.

[24] Lu J., Ju X., Qiao W., Men B., Wang R., Wu J. (2014). Azimuthally acoustic logging tool to evaluate cementing quality. J. Geophys. Eng..

[25] Lu J.-Q., Ju X.-D., Men B., Zhao H., Qiao W.-X., Duan W. (2017). An acousto-electric effect logging detector in boreholes. J. Geophys. Eng..

[26] Su Y., Fang X., Tang X. (2020). Measurement of the shear slowness of slow formations from monopole logging-while-drilling sonic logs. Geophysics.

[27] Zhang K., Ju X.D., Tan B.H., Lu J.Q., Men B.Y., Wu W.H., Chen J.Y. (2017). New excitation method for acoustic logging transmitters. J. Geophys. Eng..

[28] Lu J.Q., Ju X.D., Cheng X.Y. (2009). Transducer Firing Method for Multi-pole Array Acoustic Logging Tool. High Volt. Eng..

